# Editorial: Insights in veterinary regenerative medicine: 2022

**DOI:** 10.3389/fvets.2023.1285275

**Published:** 2023-09-20

**Authors:** Scott J. Roberts

**Affiliations:** Department of Comparative Biomedical Sciences, The Royal Veterinary College, London, United Kingdom

**Keywords:** regenerative medicine, stromal cells, platelet rich plasma, extracellular vesicles, translational research

The progress of the veterinary regenerative medicine field over the first two decades of the twenty-first century has been considerable. Due to the similarities in many degenerative diseases (e.g., osteoarthritis; OA) between veterinary species and humans, this area of research is well suited to applying a “One Health” approach to innovations in veterinary regenerative medicine toward human medicine and *vice versa*.

In this Research Topic we aim to capture a snapshot of the research conducted, along with the questions, solutions and challenges in veterinary regenerative medicine over the preceding year. From the nine published articles in this Research Topic it is clear that current trends in veterinary regenerative medicine are synonymous with research in this area of human medicine, with a major research interest in mesenchymal stem/stromal cells (MSCs) and platelet rich plasma (PRP) for degenerative disease treatment. Interestingly, the review by Penning and van den Boom details the expanding field of organoid research and how this is contributing to new understanding. Herein, the authors pinpoint a lack of validated species-specific tools (such as antibodies and growth factors) as a major roadblock in extrapolation of human studies into that of veterinary species. The opportunities of companion animal organoid research are also thoroughly reviewed with respect to disease modeling, precision medicine and organ transplantation/replacement. The concept of standardization in MSC research and equivalence between human and veterinary research is also approached in the opinion article by Guest et al., whereby guidelines for reporting research involving the use of MSCs in veterinary settings are proposed. The minimal criteria suggested by the authors are closely aligned to the minimal criteria for defining human MSCs, set out by the ISCT (International Society for Cellular Therapy). However, the authors do propose further measures such as immunomodulatory analysis and the introduction of a Clinical Indications Prediction (CLIP) scale for veterinary MSCs, thus improving reproducibility and robustness.

The original research articles can be classified into those using MSCs or their derivatives toward regenerative applications, and those using PRP. Kearney et al. investigated whether the secretome of allogenic MSCs had anti-inflammatory effects in an equine model of joint inflammation when introduced intraarticularly. Interestingly, no differences were observed between the treatment effects of the MSC secretome and MSCs, thus if the effects are proved to be beneficial this may provide an effective off-the-shelf treatment for inflammatory joint disease in the horse. Clarke et al. took the innovative approach of investigating secreted extracellular vesicles (EVs) in the context of equine OA, where changes in the proteome of the synovial fluid EVs following integrin α10-positive MSC administration were measured. The author's data suggest that MSC-derived EVs may play a role in mediating MSC efficacy and identify specific targets that warrant further investigation. Furthering the concept of the paracrine action of MSCs, Caruso et al. show that conditioned media from equine bone marrow MSCs promote wound healing *in vitro*. However, this effect was not translatable to equine cutaneous wound healing *in vivo* upon implantation of intact MSCs. Koch et al. attempted to understand changes in cytokine composition in a surgical model of equine tendon injury using an ultrafiltration technique. From this insight, the authors reported that MSC priming with the predominant cytokine (IL-1β) at similar concentrations found *in vivo* increased expression of IL-6, VEGF, and PGE2; which the authors predict may confer enhanced therapeutic potential. Armitage et al. investigated the efficacy of autologous MSCs for efficacy in chronic degenerative musculoskeletal conditions in dogs, with outcome measures spanning joint mobility through to pain. In this study, MSCs were introduced alone or in combination with PRP. The authors conclude that the treatments resulted in positive effects in the patient's status, indicating the utility of MSC treatment toward multiple degenerative musculoskeletal conditions. This study does highlight the potential of PRP as an adjuvant for MSC therapy, however, two other papers in this Research Topic detail the clinical application of PRP alone in canines. Matos Cruz and Mason report on their study where PRP was used following bilateral arthroscopic subtotal coronoidectomy of the fragmented medial coronoid process, with owner-reported outcomes recorded as a measure of efficacy. The authors conclude that when surgery was not successful, PRP appeared to reduce lameness long term. Successful outcome of PRP treatment was also reported in a case study of Flexor carpi ulnaris tendinopathy from Franini et al.. The authors report that their PRP-associated treatment programme resulted in the resolution of patient lameness and improvements in tendon parameters.

Although limitations exist with each of the studies detailed herein (discussed within each publication), this Research Topic showcases the advances the field is currently experiencing in the translation of basic research towards clinical application. Furthermore, it highlights the technological and reporting advances the field requires to ensure veterinary regenerative medicine can keep pace with advances in the human arena. Only through the creation of species-specific and validated reagents will we be able to realize this goal. Increased technological possibilities, such as single cell transcriptomics, epigenetic analysis and spatial technologies, frequently used in the investigation of human regenerative solutions, have a financial implication that is often challenging within the current funding landscape. As such, we need to make better use of comparative analysis and *in silico* tools to extrapolate data from all other fields and species.

Adoption of these concepts will contribute to achieving a successful “One Health” future for regenerative medicine.

## Author contributions

SR: Writing—original draft, Writing—review and editing.

